# Could Periodontal Disease through Periopathogen *Fusobacterium nucleatum* Be an Aggravating Factor for Gastric Cancer?

**DOI:** 10.3390/jcm9123885

**Published:** 2020-11-29

**Authors:** Petra Șurlin, Flavia Mirela Nicolae, Valeriu Marin Șurlin, Ștefan Pătrașcu, Bogdan Silviu Ungureanu, Andreea Cristiana Didilescu, Dan Ionuț Gheonea

**Affiliations:** 1 Department of Periodontology, University of Medicine and Pharmacy of Craiova, 200349 Craiova, Romania; surlinpetra@gmail.com; 2 Department 1st of Surgery, University of Medicine and Pharmacy of Craiova, 200349 Craiova, Romania; stef.patrascu@gmail.com; 3 Department of Gastroenterology, University of Medicine and Pharmacy of Craiova, 200349 Craiova, Romania; boboungureanu@gmail.com (B.S.U.); digheonea@gmail.com (D.I.G.); 4 Department of Embriology, University of Medicine and Pharmacy Carol Davila of Bucharest, 020021 Bucharest, Romania; andreea.didilescu@gmail.com

**Keywords:** periodontitis, *Fusobacterium nucleatum*, gastroenterological cancer, gastric cancer

## Abstract

Periodontal disease affects the supporting tissues of the teeth, being a chronic inflammatory disease caused by specific microorganisms from subgingival biofilm. *Fusobacterium nucleatum* is a Gram-negative anaerobic bacterium that acts as a periodontal pathogen, being an important factor in linking Gram-positive and Gram-negative bacteria in the periodontal biofilm, but its involvement in systemic diseases has also been found. Several studies regarding the implication of *Fusobacterium nucleatum* in gastro-enterological cancers have been conducted. The present review aims to update and systematize the latest information about *Fusobacterium nucleatum* in order to evaluate the possibility of an association between periodontal disease and the evolution of gastroenterological cancers through the action of *Fusobacterium nucleatum*, highlighting gastric cancer. This would motivate future research on the negative influence of periodontal pathology on the evolution of gastric cancer in patients suffering from both pathologies.

## 1. Introduction

Periodontal disease is the most common chronic inflammatory disease, and severe periodontitis affects approximately 10% of the global population, with variations by country and world region [1]. It affects the supporting tissues of the teeth and is caused by specific microorganisms, thus leading to a progressive destruction of the adjacent bone and periodontal ligament while affecting the immune cells [2]. Periodontal pathogenic bacteria from oral plaque are the main etiologic agents for periodontitis, whereas calculus, smoking, self-inflicted injuries, caries, or iatrogenic factors, such as over-contoured restorations, restorative overhangs, subgingival margins of the restorations, unpolished surfaces, or complications of orthodontic therapy are considered local predisposing factors [3]. Systemic factors that are implicated as risk indicators in periodontitis are endocrine disorders and hormonal changes, such as diabetes mellitus, hematologic disorders, and immune deficiencies, such as anemia or leukemia, smoking, medications, stress and psychosomatic disorders, other genetic disorders, and age. There are systemic conditions that are proven as being in a bidirectional relationship with periodontitis, with the most studied being diabetes mellitus. Diabetic patients have a threefold higher risk for developing severe periodontitis compared to healthy individuals, with poor glycaemic control having an important role in determining increased risk. The glycemic control may also be negatively influenced by periodontitis [4]. Other systemic conditions that interconnect with periodontitis are coronary heart disease, atherosclerosis, adverse pregnancy outcomes, chronic obstructive pulmonary disease, acute respiratory disease, and asthma [5].

On the basis of the bacterial etiology for periodontal disease, Socransky et al. first categorized the subgingival biofilm in five microbial complexes, which are colorimetrically coded as yellow, green, purple, orange, and red [6]. The first three complexes—the yellow (multiple species of *Streptococcus*), the green (*Capnocytophaga*, *Eikenella corrodens*), and the purple complex (*Veillonela parvula* and *Actinomyces odontolyticus*)—are compatible with periodontal health, while the others—the orange (*Fusobacterium nucleatum*, *Prevotella intermedia*, and *Campylobacter rectus*) and the red (*Porphyromonas gingivalis*, *Treponema denticola*, and *Tanerella forsythia*) complexes—are involved in periopathogenesis, with the red one being composed of the highest virulent and aggressive bacteria. The orange complex is strongly implicated in the progression of periodontal disease, providing structural support or serving as metabolic cornerstones. This complex actually links the primary colonizers from the first three complexes, to the secondary colonizers: Gram-negative and anaerobic microbial species. Without this orange complex, the aggressive bacteria from the red complex are not capable of surviving in the human oral microbiota [7,8]. *Fusobacterium* binds to all oral bacteria, while other bacteria only bind to streptococci or other species [9].

*Fusobacterium nucleatum* has received more attention recently, as multiple studies suggest its implication not only in periodontal disease but in multiple systemic conditions, such as cardiovascular diseases, adverse pregnancy outcomes, gastro-intestinal disorders, rheumatoid arthritis, or diabetes [10]. A recent study showed that the same strains of *Fusobacterium* from the oral cavity were found grafted on colorectal cancer (CRC) tissues, thus suggesting the oral dissemination of the bacteria [11]. There are numerous studies about *Fusobacterium* and its implications in CRC, but for gastric cancer, the information is yet to come.

Gastric cancer is ranked fifth among all cancers, both in terms of incidence and mortality, with all ages and genders included. More than 1 million new cases are diagnosed each year and it is estimated that 783,000 people will die from the disease every year [12], representing 8.2% of all cancer deaths. There is a huge variation in incidence and mortality between regions all over the globe, with the highest incidence being reported in Asian countries such Japan, China, and South Korea, and the lowest in North America and northern Europe. Moreover, the highest death rates are recorded in Turkmenistan, Kirghizstan, and Iran [12].

There is a global tendency for the incidence rates to decrease constantly in the last decades, a trend that will continue [13]; however, in Romania, the gastric cancer incidence has still been quite high over recent years, presenting variations across the country. It is more common in men compared with women, reaching a peak of incidence around age of 60. Gastric cancer mortality in the world places Romania among the countries with average mortality [12,14]. In Japan and Australia, the mortality is decreasing, in contrast to the USA where, in spite of a fall in incidence, the average survival rate after 5 years is only 31%, mainly because of advanced stage at the moment of diagnosis [15]. Overall, men are involved twice more often than women [12]. Younger people, under 35 years are rarely affected, but the disease has a more aggressive course, indicating a potential genetic predisposition. Almost 95% of the gastric cancers are adenocarcinoma with two histologic subtypes, intestinal and diffuse, according to Lauren classification [16].

On the basis of the anatomic sites, gastric adenocarcinomas can be divided as cardia and non-cardia or distal stomach cancer [17]. There are some etiopathogenic differences between distal and cardia gastric cancers. Distal stomach cancers are a consequence of chronic gastritis; inflammation of the gastric mucosa; and the environmental factors, such as *Helicobacter pylori (H. pylori)*, dietary habits, and low socioeconomic status. Cardia gastric cancers are more related to obesity or gastroesophageal reflux disease [18]. Mutations of certain genes, inherited from parents, or familial adenomatous polyposis have been found to increase the risk for gastric cancer [19]. Other incriminated factors include smoking, heavy alcohol drinking, chemical exposure, dietary habits, obesity, pernicious anemia, gastric surgery, radiation, and Epstein–Barr virus [16].

The main risk factor for stomach cancer remains *H. pylori*, being known that 90% of the cases of distal stomach cancers are related to it [17]. Moreover, some studies show that by eradiction of *H. pylori*, the prevalence for non-cardia cancers dropped [16]. 

A recent study found high levels of *Fusobacterium* and Clostridium in patients with gastric cancer, while the levels of *H. pylori* were barely represented. This is thought to create a niche microenvironment for other secondary bacteria, which will replace *H. pylori* as the predominant species and trigger a more aggressive oncogenesis. The gastric microbiota has a high number of passenger bacteria, which travel from the oral cavity to the gut, besides its normal, resident bacteria [20]. Multiple species of Fusobacteria, Porphyromonas, Prevotella, Klebsiella, Neisseria, Rothia, Veillonella, and Streptococcus have been identified to this date, with the composition of the gastric microbiome varying widely with dietary habits [20,21]. High levels of *Fusobacterium nucleatum*, *Clostridium colicanis*, and Lactobacillus may be considered as a bacterial signature for gastric cancer, suggesting that *Fusobacterium* may play an active role in gastric oncogenesis, without forgetting its demonstrated role in CRC [20]. *Fusobacterium nucleatum* and *Clostridium colicanis* have 100% sensitivity and could be considered viable diagnostic markers for early diagnosis of gastric cancer, along with biopsies through upper gastro-intestinal tract endoscopic examinations, which are considered an important method for the screening of the patients [20,22].

Several studies regarding the implication of *Fusobacterium nucleatum* in gastro-enterological cancers have been made, starting with the discovery of FadA adhesin (*Fusobacterium* adhesin A) [23] and continuing with its involvement in CRC [11,24,25,26,27]; some studies have highlighted the link between *Fusobacterium nucleatum* and gastric cancer [20,25,28]. 

### Aim

Starting from the fact that *Fusobacterium nucleatum* is one of the most important bacterium in the evolution of periodontal disease, with its involvement in some types of gastroenterological cancer having been proven, the present review aims to update and systematize the latest information about *Fusobacterium nucleatum*, its pathogenicity, the oncogenic mechanisms, and its implications in oncological diseases, aiming to draw attention to the fact that there are sufficient data to support the possibility of an association between PD and the evolution of these cancers through the action of *Fusobacterium nucleatum*, highlighting gastric cancer. This would motivate future research on the negative influence of periodontal pathology on the evolution of gastric cancer in patients suffering from both pathologies. The results of these studies could lead to important conclusions for periodontal and gastroenterological practice, such as the possibility of reducing the risk of negative evolution of gastric cancer by performing periodontal screening of this type of cancer patient and periodontal treatment where appropriate and improving the quality of life of these patients.

## 2. *Fusobacterium nucleatum*

### 2.1. Involvement in Periodontal and Systemic Diseases

*Fusobacterium nucleatum* is present in oral health but it has also been found in periodontal changes from gingivitis to severe forms of periodontitis. It has been found more abundantly in deep periodontal pockets, in places with high inflammation, and in more severe cases [29].

*Fusobacterium* links Gram-positive and Gram-negative bacteria in the periodontal biofilm and establishes conditions for the anaerobes. It is commonly found to a higher extent in periodontal sites, but according to some authors, it is not responsible for the progression of bone and attachment loss in periodontitis [30]. Periodontal bone loss and abscess were reported in mice infected only with *Fusobacterium nucleatum* but with no other bacterial stain [31].

Its titer varies according to environmental factors, with higher levels found in smokers [32] and in chronic periodontal patients with uncontrolled type 2 diabetes [33]. *Fusobacterium nucleatum* is also present in young adults without clinical signs of gingivitis nor periodontitis, but that is the witness of a dysbiotic microbiota [34]. Besides the oral sites, it can often be found in infections in the rest of the body such as the brain, lungs, and abdominal organs, or in intrauterine infections [30]. It is also associated with head and neck infections, such as otitis, sinusitis, mastoiditis, and peritonsillar and retropharingeal abscesses, as well as in joint and bone infection [35]. Moreover, it seems that *Fusobacterium nucleatum* has numerous implications in preterm premature rupture of membranes, low birth weight, neonatal sepsis, and preterm labor [36]. Furthermore, there are reports about term stillbirth caused by oral *Fusobacterium*, indicating that bacteria from the placenta and fetus were translocated from the maternal subgingival plaque and led to acute inflammation and fetal death [37]. *Fusobacterium* has been linked to numerous gastro-intestinal disorders, including appendicitis, inflammatory bowel disease (IBD), and CRC [38,39,40,41]. It has been identified in ruptured cerebral aneurysm or atherosclerotic plaques [42,43]. *Fusobacterium nucleatum* is also involved in rheumatoid arthritis, Alzheimer’s disease, and diabetes [44,45,46].

### 2.2. Mechanisms of Pathogenicity of Fusobacterium nucleatum

*Fusobacterium nucleatum* stimulates the production of interleukin-8 (IL-8) from the epithelial cells, thus increasing the local inflammation; it suppresses T cell responses and upregulates the apoptosis of the peripheral white blood cells. It also stimulates the production of human B defensins and innate antimicrobial peptides in gingival epithelial cells, thereby suppressing the growth of the other competitive bacteria [23,47,48,49,50].

*Fusobacterium nucleatum* is highly susceptible to cytokines and phagocytosis. It induces a weak immune response, but in some conditions, Fusobacteria can trigger an inflammatory response by raising the levels of the proinflammatory cytokines and metalloproteinases (MMPs). Likewise, it upregulates IL-8, MMP9, the production of collagenase 3 (MMP13), and other proteolytic enzymes (MMPs). Increased cell migration and survival of the infected epithelial cells are determined by MMP13 [30,51]. *Fusobacterium nucleatum* produces serine proteases that destroy elements of the periodontal connective tissue such as the extracellular matrix proteins, fibrinogen, fibronectin, and type I and IV collagen. Finally, the proteases affect the host defense system, mainly involving the complement and the IgA, but not IgG, thus helping the bacteria to escape the host’s immune system. It does not have a high proteolytic activity, as *Fusobacterium* is often found in association with more powerful bacteria [30].

Another role of this bacterium is the impairment of immune function, as well as other bacterial stain growth, but those are part of Socransky’s red complex. It triggers apoptosis of the polymorphonuclear cells or the mononuclear cells from the peripheral blood, while in lymphocytes it induces aggregation and apoptotic cell death. It also downregulates the functions of T cells and B cells [30].

Bacteria from Socransky’s complexes such *Prevotella intermedia* will always be detected in periodontal disease together with *Fusobacterium nucleatum*, but never alone, and combinations of *Fusobacterium*, *Bacteroides forsythus*, and *Campylobacter rectus* have been found in deep pockets that are refractory to treatment. Only *Fusobacterium nucleatum* and *Aggregatibacter actinomycetemcomitans* can trigger programmed death of the peripheral blood mononuclear cells (PBMCs), and *Fusobacterium* kills more polymorphonuclear neutrophils (PMNs) than PBMCs. Fusobacteria kills the immune cells from the first line and helps other pathogen bacteria to bind, and thus periodontal disease starts to develop [30,48].

Chemokines that attract neutrophils, monocytes, and cytokines, which in turn trigger the adaptive immune system, are produced by the cells of the epithelium in response to the bacteria. Antimicrobial peptides, such as β-defensins, α-defensins, and cathelicidine, maintain the balance between disease and health. *Fusobacterium nucleatum* stimulate the synthesis of multiple protease inhibitors, which modifies the proteases released by the neutrophils, and thus it can control the tissue damage, acting as a protective response against the pathogenic bacteria such as *Porphyromonas gingivalis*. *Fusobacterium* was identified in uninflammed tissues, given that it upregulates chemokines and cytokines (IL-8) and down-regulates NF-kB (nuclear factor kappa-light-chain-enhancer of activated B cells), a protein complex that controls the transcription of DNA and cytokine production [30]. *Fusobacterium nucleatum* upregulates the synthesis of butyrate, which is a cytotoxic fatty acid involved in periodontal disease, especially in aggressive periodontitis [52,53].

### 2.3. Mechanism of Adhesion of Fusobacterium nucleatum Through FadA

*Fusobacterium nucleatum* has an excellent adherence as it binds to eukaryotic and prokaryotic cells as well as to extracellular macromolecules due to its adhesins. FadA, an adhesin discovered in 2005, makes a bond with the KBcells of the oral mucosa through the surface proteins. It can be found in two forms, intact and secreted, only in oral species of *Fusobacterium*: *Fusobacterium nucleatum*, *Fusobacterium periodonticum*, and *Fusobacterium simiae*. The other extra-oral species do not own this special adhesin, and this special feature provides an efficient marker in detecting *Fusobacterium* in other organs and indicating the source of infection. FadA does not resemble any known adhesin and is not essential for the integrity of the bacteria; however, it is fundamental for binding to host cells [23].

FadA is composed of 129 amino acid residues, with a signal peptide consisting of 18 amino acids. It has two forms: the intact preFadA, localized in the inner membrane, and the secreted mature FadA (mFadA), outside of the bacteria. Pre-FadA is insoluble, while mFadA is soluble at neutral pH. mFadA alone cannot bind to epithelial cells, but if it is mixed with preFadA, binding and invasion may also occur. The preFadA–mFadA complex starts from the inner membrane and extends through the outer membrane [54].

A correlation between FadA gene and gingival inflammation has been made. Higher expression was found in patients with gingivitis, periodontitis, and, to a lesser degree, in patients with gingival inflammation during orthodontic treatment. It has been stated that Fusobacteria without the FadA gene may have a decreased pathogenicity in comparison with the strains displaying FadA, while only *Fusobacterium nucleatum* and *Fusobacterium periodonticum* encode FadA [10,23,55]. FadA has lower levels in the dental plaque samples of periodontal healthy patients, and its levels start to rise within the periodontal inflammation [55].

Cell junction molecules, the cadherins, are linked by FadA. The adhesin binds to E-cadherin (a cell adhesion molecule) on CRC and epithelial cells and to vascular endothelial cadherin (VE-cadherin) on the endothelial cells, and it may be the reason why it is present in so many body sites and tissues [10,56,57]. When binding to endothelial cells, it makes the junctions get loose, and thus the permeability of the endothelial layer increases. Therefore, FadA facilitates peri-cellular invasion and direct invasion into the host cells, with this potentially being the mechanism for systemic dissemination [57]. Moreover, with the permeability rising, bacteria from the surrounding tissue can pass more easily. A permanent homeostasis is present between the pro- and anti-inflammatory factors, but once *Fusobacterium nucleatum* gets outside of the oral cavity, dysbiosis occurs, an increased inflammation appears, and *Fusobacterium* is turned into a real pathogen [10].

## 3. *Fusobacterium nucleatum* and Carcinogenesis

### 3.1. Fusobacterium nucleatum and Oral Cancer

Several bacteria have been incriminated as etiologic factors for oral carcinogenesis. Some of them are periodontal pathogens, such as *Fusobacterium nucleatum*, *Treponema denticola*, and *Porphyromonas gingivalis* [24]. Carcinogenesis mechanisms of *Fusobacterium* are chronic infection, suppression, and evasion of the immune system, and the interaction of cell surface molecules of the bacteria, such as Fap2, FadA, and LPS, with stromal cells and immune system [58]. It has been demonstrated that oral squamous cell carcinoma (OSCC) could be linked to *Fusobacterium nucleatum* and other bacteria, such as *Escherichia coli*, *P. gingivalis*, and *Streptococcus mitis* [58,59]. In salivary and tissue samples of the patients with oral and head and neck squamous cell carcinoma, researchers found higher levels of *Fusobacterium nucleatum* [24,60,61,62,63,64].

One of the mechanisms of the carcinogenesis of oral Fusobacteria is chronic inflammation. The bacteria upregulates the production of inflammatory cytokines and chemokines, triggering an inflammatory environment that favors tumor progression [58,65]. The expression of inflammatory genes and oncogenes is activated by the E-cadherin/β-catenin signaling pathway, which is upregulated by FadA [24,56].

Another etiologic factor for carcinogenesis is that Fusobacteria help cell proliferation, migration, and survival. The bacteria trigger the production of MMP-9 and MMP-12 in epithelial cells. These are important factors for the invasion and metastasis of the oral tumor [24,51,59].

Another role for *Fusobacterium* in carcinogenesis is its immunosuppressive role. It impairs natural killer (NK) and T cell functions [66] and downregulates the NK cell’s cytotoxicity and the response of T cells through the interaction between Fap2 protein and T cell immunoglobulin [67]. *Fusobacterium* is capable of inducing cell apoptosis for the polymorphonuclear neutrophils (PMNs) and the peripheral mononuclear blood cells (PMBCs) [48]. Very recently, it was stated that an increase of *F. nucleatum* is frequently observed in OSCC tissues compared to healthy oral mucosa, with detection of *F. nucleatum* being correlated with the clinical stage of oral cancer. Although the detailed mechanism is still unclear, *Fusobacterium* species have been suggested to be associated with cell adhesion, tumorigenesis, epithelial-to-mesenchymal transition, inflammasomes, and cell cycle in oral cancer. It has also been suggested that oral hygiene management for reducing the amount of *Fusobacterium nucleatum* may contribute to the prevention of OSCC [68].

By knowing which bacteria play which role in cancer, we can develop new methods for early detection, leading to a reduced morbidity and mortality rate. By removing the bacteria that cause immunosuppression, researchers can develop new therapeutic strategies [24]. There have been multiple studies about therapies for cancer by targeting the bacteria, such as vaccination, change of diet, and the use of probiotics [69,70,71,72,73].

### 3.2. Fusobacterium nucleatum and Gastroenterological Cancer

There are bacteria and viruses associated with about 20% of total cancer incidence [25,74]. *Fusobacterium* provides structural support for other microorganisms, as it can bind up to 10 *Streptococcus sanguis* and it is considered as a strongly implicated factor in the polymicrobial biofilms [26,75].

It has been discovered recently that oral *Fusobacterium nucleatum* colonizes colorectal adenocarcinomas, being responsible for tumor progression and evasion of the immune system [66]. CRC patients have higher levels of *Fusobacterium nucleatum* than the normal population [26,76]; the higher the levels, the worse the prognosis, while the resistance to chemotherapy and recurrence are increased [26,77,78]. The presence of *Fusobacterium* also increases the metastatic rate [26], and it also plays an important role in premalignant lesions of the colon [79]. Cellular proliferation is stimulated and an inflammatory environment favorable for the progression of the tumor is created [60,80,81]. Higher levels of *Fusobacterium nucleatum* are correlated with a poor prognosis for CRC [38], and thus further treatment options based on the elimination of the bacteria, as well as possibly the D-galactose–β(1-3)-N-acetyl-D-galactosamine (Gal-GalNAc) complex, should be considered [25]. By introducing metronidazole in the therapeutic scheme for the CRC patients, the growth of the tumor and the levels of Fusobacteria were reduced, but antibiotics also inhibit other bacterial species, probably causing dysbiosis [26].

Oral Fusobacteria recognize the CRC through the lectin Fap2. This lectin finds on the surface of the tumor the receptor Gal-GalNAc and establishes a connection. The metastases are also colonized by *Fusobacterium nucleatum*, because of the higher Gal-GalNAc levels. High Gal-GalNAc levels were found in tumors of epithelial tissue with glandular origin and in adenocarcinomas of the stomach, colon, pancreas, breast, esophagus, lungs, etc. However, in the stomach and cervix, there were no significant differences regarding the Gal-GalNAc levels between the normal and cancer samples; moreover, for the non-adenocarcinoma tumors, the levels were also similar [25,82].

RadD protein is another adhesin of the bacteria localized on the outer membrane. It helps Fusobacteria to connect with *Streptococcus mutans* [26,83] or with *Candida albicans* [84]. FadA binds to the VE-cadherin and triggers B-catenin signaling, stimulating inflammatory responses. The transcription factors, such as nuclear factor-kB (NF-kB), are up-regulated, which in turn will increase the tumoral cell proliferation in CRC [85,86].

The tumor microenvironment is modified by *Fusobacterium nucleatum* through tumor-associated neutrophils (TANs) and macrophages (TAMs), as well as myeloid-derived suppressor cells (MDSCs) [27]. By releasing RNA into the cytoplasm of the host cell, the bacteria activate oncogenes and inflammatory genes [87,88]. Moreover, it easily penetrates into the bloodstream because of FadA’s binding to VE-cadherin, which increases endothelial permeability [57].

Neutrophils and macrophages that produce nitric oxide (NO) will begin to gather at the inflammatory site through the tumor necrosis factor alpha (TNF-α), IL-8, and other chemokines. Nitric oxide will lead to oxidative stress into the stromal and epithelial cells, which will modify the structure of DNA by damaging it [27]. The levels of certain interleukins (TNF-α, IL-6, IL-8, and IL-1β) found in higher levels in tissues with inflammation by activation of NF-kB-driven inflammation [60,89], are correlated with FadA’s expressions in CRC tissues [56].

Identical strains of *Fusobacterium nucleatum* were found in saliva and colorectal carcinoma tissues, from stages 0 to 4, thus suggesting the oral dissemination of the bacteria [11], with frequent bacteriemia in patients with periodontitis helping Fusobacteria’s hematogenous translocation to other tumor sites [25]. *Fusobacterium nucleatum* was detected in 20% of the tissues with esophageal cancer, 10% of the gastric cancers, and 45% of the CRC tissue by using polymerase chain reaction (qPCR). Higher levels were found in superficial areas of the esophageal tissues, suggesting that the bacterium probably favors the growth of the tumor as it cannot infiltrate into the invasive area. *Fusobacterium* was also detected in healthy adjacent tissues for esophageal cancer and CRC [28].

Under pathogenic conditions, some specific bacteria become predominant in the microbiome and can trigger or even contribute to the progression of the disease. *H. pylori* is one of those bacteria, as its role in gastric cancer has been widely demonstrated [20,90,91].

#### Involvement of Bacteria in Gastric Cancer

*H. pylori* has been incriminated in the etiology of gastric cancer [92,93,94]. However, its level decreases in gastritis patients, and half of those are tested negative for these bacteria [95]. Only 1–2% of those that were tested positive will develop gastric cancer, and with the evolution of the disease, *H. pylori* will become undetected [96].

The composition of the gastric microbiome varies very easily with dietary habits, with multiple species of *Fusobacterium*, Porphyromonas, Prevotella, Klebsiella, Neisseria, Rothia, Veillonella, and Streptococcus having been identified at this level [21]. Compared to the intestinal microbiome, the gastric mucosa is less complex because of its thickness and acidity, while most of the bacteria lay in the gastric juice [97]. The microbiota of the gastric epithelium of patients with gastritis resembles the one from patients with intestinal metaplasia, whereas the one from patients with gastric cancer is more different [20].

The importance of *H. pylori* in gastric lesions comes from the fact that it can pass through the mucus layer. There, the gastric mucosa are colonized and the infection occurs, potentially lasting a long time because of the local conditions. The bacteria raise the pH and produce urease, modifying the micro-environment [20,94]. *H. pylori* alters the functions of the cells of the gastric mucosa and triggers a strong inflammatory response [98]. The cell migration is increased while the apoptosis is downregulated. The mucosal integrity is disrupted through a complex mechanism, and all of these events favor the transformation of the cells of the gastric mucosa and can increase the risk of gastric cancer [99]. The interaction between *H. pylori* and the gastric microbiota may be a crucial step in gastric carcinogenesis [100]. It is thought that it creates a niche microenvironment for other secondary bacteria, which will replace *H. pylori* as the predominant species and trigger a more aggressive oncogenesis [21].

In *H. pylori*-negative patients, the gastric microbiota was more diverse, with species such as Prevotella, Veillonella, Neisseria, Campylobacter, Sphingomonas, and Streptococcus, many of them probably transiting to the lower digestive tract. A recent study found no significant differences between the cancer and non-cancer groups. High levels of *Fusobacterium* and Clostridium were determined in patients with gastric cancer, while those of *H. pylori* were barely represented. In gastritis, *H. pylori* can represent more than 90% of the gastric microbiota, while in gastric cancer patients, none of them showed an extreme dominancy of the bacteria. Specific bacteria, such as *Fusobacterium*, Clostridium, and Lactobacillus, were identified in almost half of the cancer group. Lactobacillus was found in lower levels than Clostridium and *Fusobacterium*. This study suggests that *Fusobacterium* may play an active role in gastric oncogenesis. The species of *Fusobacterium* were found in the cancer group and in the non-cancer group, but in lower abundance, while the species of Lactobacillus and Clostridium were found only in the cancer group [20]. The most abundant species of Clostridium found in gastric cancer samples was *Clostridium colicanis* [20,101,102].

A drastic change in the gastric microbiota takes place in gastric cancer with regards to the presence of Lactobacillus as a high-abundance bacterium. The most abundant species detected in gastric cancer samples were *Fusobacterium nucleatum*, *Fusobacterium canifelinum*, *Clostridium colicanis*, *Lactobacillus reuteri*, and *Lactobacillus gasseri* [103,104,105].

The species *F. nucleatum*, *F. canifelinum*, *L. reuteri*, *L. gasseri*, and *C. colicanis* were defined as a cancer-associated signature, thus generating a mean for discrimination between cancer and non-cancer groups. By restricting this to three species—*F. nucleatum*, *F. canifelinum*, and *C. colicanis*—the level of accuracy decreases, but these three species were found in more than half of the cases with gastric cancer. *Fusobacterium nucleatum* and *Clostridium colicanis* have 100% sensitivity and can successfully identify gastric cancer, potentially being considered diagnostic markers for early diagnosis [20]. Other studies indicate potentially important roles of *Peptostreptococcus stomatis*, *Dialister pneumosintes*, *Slackia exigua*, *Parvimonas micra*, and *Streptococcus anginosus* in gastric cancer progression [106].

There might be connections between intestinal cancers (such as gastric and CRC) and oral infection diseases, as well as between the gut and oral microbiome [27]. Even though *Fusobacterium* nucleatum appears as a key pathogen, it is the symbiosis of the microbiota that remains as the main element of oral and systemic health. There are studies suggesting different screening methods for colorectal cancer and polyp detection, pointing towards the assessment of the oral microbiota as an efficient detection tool for early stages of gastro-intestinal premalignant and malignant diseases [107,108].

## 4. Periodontal Disease and Cancer

Periodontal disease was found to increase the risk of oral cancer by nearly twofold [109]. There are data showing that periodontitis was independently associated with OSCC; thus, the risk of OSCC could be modulated by reducing periodontitis [110].

In a recent publication that explored the role of chronic inflammation as a biologically plausible mechanism that links periodontitis and risk of cancer, there are highlighted studies that have examined the potential importance of certain periodontal pathogens in this association, such as *Porphyromonas gingivalis* and *Fusobacterium nucleatum*. There are data from epidemiological evidence on periodontal disease and cancer risk that point to a positive association. It appears that risk may be higher in close proximity to the oral cavity such as esophagus and upper gastrointestinal tract. The role of periodontal disease and oral microorganisms needs to be studied further with regards to their association with cancer risk [9,111].

Genetic and molecular studies can explain the high risk of development of cancers from pre-existing inflammatory lesions such as those from periodontitis. Due to high prevalence of periodontitis and the risk of developing of different cancers including oral cancer, diagnostic and therapeutic strategies for periodontal treatment should be considered [112].

Periodontal chronic infection was found to be positively associated not only with risk of oral cancer but also with lung and pancreatic cancers, with additional prospective studies being necessary in order to better investigate the strength of these associations [113].

In a cohort study based on 68,273 adults followed for 10 years, the results showed stronger associations of periodontitis with increased overall cancer mortality. A higher pancreatic cancer mortality among patients with periodontitis contributed considerably to the difference in overall cancer mortality, but this difference was not due only to pancreatic cancer deaths alone [114].

Women with periodontitis have a significantly higher risk of breast cancer, with *Fusobacterium nucleatum* being involved in breast tumor growth, and men with periodontitis have a significantly higher risk of prostate and hematological cancers. Periodontitis might be associated with increased cancer risk, particularly with hematological, breast, and prostate cancers [115,116].

It has also been suggested that oral hygiene management for reducing the periodontal biofilm and consequently the amount of *Fusobacterium nucleatum* may contribute to the prevention not only of OSCC but also CRC. The authors also suggested investigating whether the mechanism of *Fusobacterium nucleatum* in OSCC overlaps with that of CRC [68]. Moreover, it was shown that the reduction of *Fusobacterium nucleatum* through oral prophylaxis leads to the establishment of a symbiotic microbiota [117].

The studies regarding the relationship between the gastroenterological cancers and periodontitis are limited; for certain types of these cancers, the results are more consistent in finding a link between periodontal disease and cancer mortality risk [114,118]. For gastric cancer, the findings are inconsistent; a retrospective cohort study on a population from Taiwan, did not observe a significant association between the severity of chronic periodontitis and the risk of gastric cancer [119].A previous study reported no association of periodontal disease with this type of cancer in male health professionals [120].

In case-control studies, the authors found that smoking, gingival inflammation, and clinical attachment loss in periodontitis patients were statistically significantly different between patients who had gastric cancer and healthy individuals [121], with clinical attachment loss as an index for periodontal disease being statistically significantly associated with the risk of gastric cancer development [122].

All these studies, retrospective cohort and case-control, have limitations that should be considered when interpreting the results, but these controversial results motivate further studies to investigate the possible association between the presence and severity of periodontal disease and the evolution of gastric cancer, evaluating not only the clinical parameters but also the colonization with *Fusobacterium nucleatum* of the gastric tumor tissues and the surrounding healthy tissues.

## 5. Conclusions

Scientific data support the possibility of an association between periodontal disease and the evolution of gastro-enterological cancers through the action of *Fusobacterium nucleatum*. This would motivate future research on the negative influence of periodontal pathology on the evolution not only of colorectal but also gastric cancer. The results of these studies could be important for periodontal and gastroenterological practice in terms of reducing the risk of negative evolution of gastric cancer through periodontal screening and periodontal treatment where appropriate, improving the quality of life of these patients.
